# The Prevalence and Characteristics of IgA Antibodies to β2-Spectrin and CBX3 in Immunoglobulin A Nephropathy

**DOI:** 10.1016/j.ekir.2025.02.025

**Published:** 2025-03-03

**Authors:** Ayako Koizumi, Yoshihito Nihei, Kazuaki Mori, Ryousuke Aoki, Hitoshi Suzuki, Jonathan Barratt, Yusuke Suzuki

**Affiliations:** 1 Department of Nephrology, Faculty of Medicine, Juntendo University, Tokyo, Japan; 2 Department of Nephrology, Juntendo University Urayasu Hospital, Juntendo University, Chiba, Japan; 3 Department of Cardiovascular Sciences, Leicester General Hospital, University Hospitals of Leicester, NHS Trust, Leicester, UK

**Keywords:** anti-CBX3 IgA antibody, antimesangium IgA antibody, anti-SPTBN1 IgA antibody, autoimmune glomerulonephritis, IgA nephropathy

## Abstract

**Introduction:**

In IgA nephropathy (IgAN), the mechanism of IgA-containing immune complexes deposition in the glomerular mesangium had been unclear. We recently reported the presence of IgA antibodies with specificity for mesangial cells (antimesangium IgA antibodies) in sera from patients with IgAN, and identified β2-spectrin (SPTBN1) and CBX3 as target antigens. However, the role of antimesangium IgA antibodies in human IgAN is unclear.

**Methods:**

We measured serum anti-SPTBN1 and anti-CBX3 IgA levels in patients with IgAN (*n* = 119) and other kidney diseases (disease control [DC], *n* = 51) using 2 independent cohorts, 1 from Japan and 1 from the UK. The study also assessed the surface expression of the autoantigens on human mesangial cells and the pattern of *O*-glycosylation of serum anti-SPTBN1 and anti-CBX3 IgA antibodies.

**Results:**

Overall, 30 and 3 patients with IgAN and DC, respectively, had detectable anti-SPTBN1 IgA antibodies (sensitivity, 25%; specificity, 94%); whereas 48 and 3 patients with IgAN and DC, respectively, had detectable anti-CBX3 IgA antibodies (sensitivity, 40%; specificity, 94%). In total, 62 patients (52%) with IgAN had detectable anti-SPTBN1 and/or anti-CBX3 IgA antibodies. The expression of SPTBN1 and CBX3 on the surface of human mesangial cells was confirmed by immunofluorescence (IF) microscopy. Serum anti-SPTBN1 and anti-CBX3 IgA antibodies from patients with IgAN were recognized by an antigalactose-deficient IgA1 antibody (KM55) by Western blotting.

**Conclusion:**

We show that anti-SPTBN1 and anti-CBX3 IgA antibodies are detected with high specificity in patients with IgAN from Japan and the UK, and are enriched for IgA1 with poorly galactosylated *O*-glycoforms.

## Graphical abstract

**Figure:**
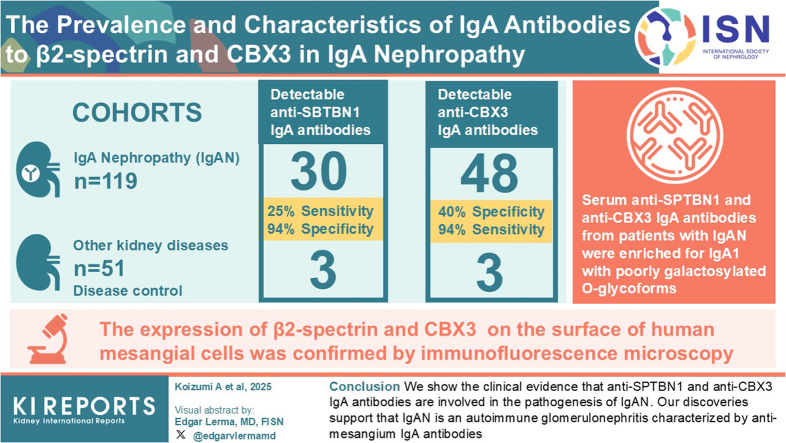

IgAN is the most common primary glomerulonephritis worldwide whose pathogenesis is commonly described within a multihit model.^1^ The first hit is an increase in serum IgA1 with *O*-glycoforms that carry fewer galactose residues at the hinge (commonly measured and reported as “Gd-IgA1”), hit 2 is the presence of IgA and IgG antibodies with specificity for these IgA1 *O*-glycoforms, which promote IgA immune complex formation (hit 3). These immune complexes are then thought to deposit in the glomerulus because of their charge and high molecular weight.^1^^,^^2^ However, in this scenario, it was unclear why immune complexes containing Gd-IgA1 selectively deposit in the mesangial region, a disease hallmark of IgAN.

The region-specific antibody deposition implies the recognition of autoantibodies against self-antigens. In several types of glomerulonephritis, circulating antibodies against glomerular cells have been identified, including anti-PLA2R antibody in membranous nephropathy (MN)^3^ and antinephrin antibody in minimal change disease.^4^ The discovery of these autoreactive antibodies has brought about paradigm shifts in our understanding of the pathogenesis, diagnosis, and treatment of the glomerulonephritis.^5^^,^^6^ It has been suggested that autoantibodies to various tissue antigens may be present in IgAN. Early studies have shown the presence of IgG, but not IgA, autoantibodies against mesangial cells in some patients with IgAN.^7^^,^^8^ Wang *et al.* reported that IgA autoantibodies against endothelial cells are present in the serum of patients with IgAN.^9^ More recently, Lafayette *et al.* showed the possibility that serum from patients with IgAN contain elevated circulating IgG or IgA antibodies to autoantigens expressed in the kidney.^10^^,^^11^ However, these reports did not sufficiently explain the exact mechanism by which IgA is selectively deposited in the “mesangial region.”

We recently reported the presence of IgA antibodies with specificity for mesangial cells (antimesangium IgA antibodies) in the sera of a murine model of IgAN, gddY mice,^12^ and patients with IgAN.^13^ We identified SPTBN1 and CBX3 as the antigens recognized by antimesangium IgA antibodies, both of which are selectively expressed on the surface of mesangial cells.^13^^,^^14^ In our previous study, serum IgA antibodies against SPTBN1 (anti-SPTBN1 IgA antibodies) and CBX3 (anti-CBX3 IgA antibodies) were respectively detected in 36% and 17% of Japanese patients with IgAN, whereas these antibodies were rarely detected in the sera of healthy Japanese individuals.^13^^,^^14^ However, the role of antimesangium IgA antibodies in human IgAN needs to be further investigated in detail. Here, we aimed to examine the prevalence and characteristics of anti-SPTBN1 and anti-CBX3 IgA antibodies in IgAN, using 2 independent cohorts, 1 from Japan and 1 from the UK.

## Methods

### Study Design, Patients, and Data Correction

Patients aged ≥ 18 years with biopsy-proven IgAN (*n* = 119) or other kidney diseases (*n* = 51, DC, Supplementary Table S1) between 1991 and 2019 were included from the Juntendo University Hospital in Japan and the Leicester General Hospital in the UK. This study was approved by the ethics review board of Juntendo University School of Medicine (M19-0223); the Northamptonshire, Leicestershire, and Rutland Ethics Committee (UHL 09873); and followed the principles of the Declaration of Helsinki. Written informed consent was obtained from all the patients. Clinical data were obtained at the time of the kidney biopsy. Blood samples were collected at the time of kidney biopsy, centrifuged, and stored at −80 °C until used. Urinary protein excretion was evaluated based on the daily amount of urinary protein (g/d).

### Histopathological Analysis

All the kidney biopsy specimens from the patients with IgAN were scored according to the revised Oxford classification criteria^15^: mesangial hypercellularity (M0: < 50% of the total glomeruli; M1: ≥ 50% of the total glomeruli), endocapillary hypercellularity (E0: absent; E1: present), segmental glomerulosclerosis (S0: absent; S1: present), tubular atrophy and interstitial fibrosis (T0: 0%–24%; T1: 25%–49%; T2: ≥ 50% of the total cortical area), and cellular or fibrocellular crescents (C0: absent; C1: 1%–24%; C2: ≥ 25% of the glomeruli).

### Plasmid Construction

A FLAG-tagged human SPTBN1 expression vector was kindly provided by Tomozumi Imamichi.^16^ The expression vector for human CBX3 with Strep-tag II at the C-terminus was generated as previously reported.^14^

### Preparation of Recombinant SPTBN1 and CBX3

Human SPTBN1 with FLAG-tag and CBX3 with Strep-tag Ⅱ were transiently expressed in human embryonic kidney (HEK) 293T cells and purified from the lysates with 1% NP-40 using the anti-FLAG M2 AffinityGel (Sigma) or Strep-Tactin Sepharose (IBA), respectively, according to the manufacturer’s instructions. FLAG-tagged SPTBN1 was eluted as previously described.^13^ Strep-tag II-tagged CBX3 was eluted with 0.1 M desthiobiotin. Purification was confirmed by Coomassie Brilliant Blue staining.

### Enzyme-Linked Immunosorbent Assays

Anti-SPTBN1 and CBX IgA antibodies were detected using indirect enzyme-linked immunosorbent assay, as previously reported with minor modifications.^13^^,^^14^ Briefly, maxisorp 384-well microtiter plates (Cosmobio, Tokyo, Japan) were coated with 30 μl of recombinant SPTBN1 (40 μg/ml) or CBX3 (10 μg/ml) overnight at 4 °C. After washing the plates 5 times with phosphate-buffered saline (PBS) containing 0.05% Tween 20, they were blocked with 80 μl of PBS containing 3% bovine serum albumin and 0.05% Tween 20 at room temperature (RT) for 2 hours. After blocking, the plates were incubated with 30 μl of diluted patient serum (1:100) at RT for 2 hours. After washing, the plates were incubated with 30 μl of goat antihuman IgA antibody conjugated with horseradish peroxidase (1:8000; Abcam, Cambridge, UK) at RT for 1 hour. After washing, bound reactants were detected by a 2-minute incubation with 3,3′,5,5′-tetramethylbenzidine. The absorbance was measured at 450 nm. We set the 99% confidence interval (mean +2.58 SD) of DC from the Japanese cohort as the cut-off value.

### Cell Culture

Primary human mesangial cells were purchased from Cell Systems Inc. They were cultured in mesangial growth media with supplements (Cell Systems Inc.) and those between passages 6 and 10 were used. HEK293T cells were cultured in Dulbecco’s modified Eagle’s medium (high glucose, with L-glutamine, and sodium pyruvate) with 10% fetal bovine serum, 100 U/ml penicillin and 100 μg/ml streptomycin.

### IF Microscopy

HEK293T cells and the primary human mesangial cells were cultured on cover glasses for 48 hours and fixed in 4% paraformaldehyde (PFA) for 10 minutes. After washing 3 times with PBS, the cells were blocked with blocking solution (DS Pharma Biomedical Co. Ltd, Dublin, Ireland) for 30 minutes and surface-stained with rabbit polyclonal anti-SPTBN1 (Abcam, 72239, Cambridge, UK) or CBX3 (Cell Signaling Technology, 2619, Massachusetts, USA) IgG antibody for 1 hour at a dilution of 1:100. After washing, Alexa Fluor 488-conjugated donkey antirabbit IgG antibody (Invitrogen, 21206, Massachusetts, USA) was added for 1 hour at a dilution of 1:300. After washing, the slides were mounted with 4′,6-diamidino-2-phenylindole (Invitrogen, Massachusetts, USA). For intracellular staining of HEK293T cells, the cells were fixed and permeabilized with PFA and 0.4% triton x-100 (for SPTBN1) or cold methanol (for CBX3). All samples were examined using an IF microscope (BZ-X710; KEYENCE).

### Purification of Serum Anti-SPTBN1 and Anti-CBX3 IgA Antibodies

We coated 384-well microtiter plates with 30 μl of recombinant SPTBN1 (40 μg/ml) or CBX3 (10 μg/ml) overnight at 4 °C. After washing the plates 5 times with PBS containing 0.05% Tween 20, they were blocked with 80 μl of PBS containing 3% bovine serum albumin and 0.05% Tween 20 at RT for 2 hours. After washing, the plates were incubated with 30 μl of the diluted serum from patients with IgAN (1:250) at RT for 2 hours. After washing, bound anti-SPTBN1 IgA or anti-CBX3 IgA antibodies were eluted with 30 μl of 0.1 M glycine HCl with pH of 3.0 and immediately neutralized with Tris. After elution, purified anti-SPTBN1 or anti-CBX3 IgA antibodies were concentrated using Amicon Ultra-4 10 K (Merck, Darmstadt, Germany). Anti-SPTBN1 and anti-CBX3 IgA antibodies could not be purified from the sera of DC patients using the same method (*n* = 3, data not shown).

### Western Blotting

Western blotting was performed to detect Gd-IgA1 and IgA, as previously reported.^17^ Briefly, purified anti-SPTBN1 or anti-CBX3 IgA antibodies were solubilized in NP-40 buffer under reducing (2 mercaptoethanol +) conditions, and boiled at 100 °C for 5 minutes; 4 ng of the purified IgA antibodies or total serum IgA was subjected to sodium dodecyl sulphate polyacrylamide gel electrophoresis, followed by immunoblotting with the following antibodies: rat anti-Gd-IgA1 antibody (KM55)^18^ as the primary antibody (1 μg/ml), antirat IgG antibody conjugated with horseradish peroxidase(1:10,000) as the secondary antibody to detect Gd-IgA1, and antihuman IgA antibody conjugated with horseradish peroxidase(1:5000) to detect total IgA.

### Statistical Analysis

Statistical analyses were performed using GraphPad Prism software version 9 (GraphPad Software, La Jolla, CA). Comparisons between > 3 groups were performed using 1-way analysis of variance. Categorical variables were presented as percentages and compared using Pearson’s chi-square and Fisher exact tests. Differences at *P* < 0.05 were considered significant.

## Results

### Sensitivity and Specificity of Anti-SPTBN1 IgA Antibodies in the Patients With IgAN

**Figure 1:**
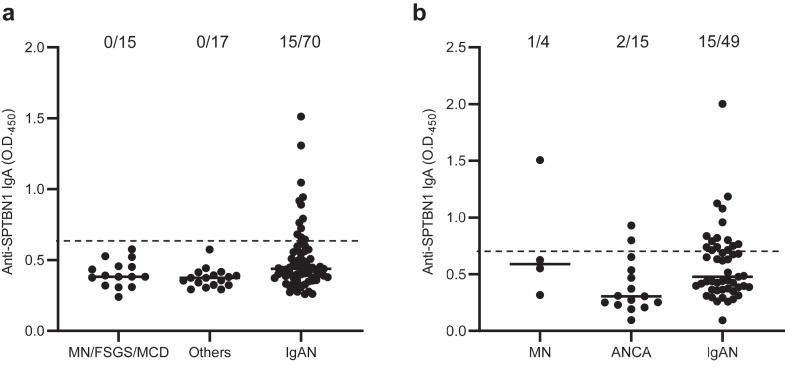
Detection of anti-SPTBN1 IgA antibodies in serum from patients with IgAN. Serum anti-SPTBN1 IgA antibody levels in patients with IgAN and DC (a) from the Japanese cohort and (b) from the UK cohort. Dashed lines show the cut-off value. Each dot represents the optical density (O.D.) value of a patient and the horizontal lines represent the medians. The numbers above the dots represent the overall number of patients with each disease and the number of patients with anti-SPTBN1 IgA antibodies. ANCA, antineutrophil cytoplasmic antibody–related vasculitis; DC, disease control; FSGS, focal segmental glomerulosclerosis; IgAN, IgA nephropathy; MCD, minimal change disease; MN, membranous nephropathy; Others, other kidney diseases; SPTBN1, β2-spectrin.

Patients with biopsy-proven IgAN (*n* = 119) and DC (*n* = 51) were recruited from Japan (IgAN = 70, DC = 32) and the UK (IgAN = 49, DC = 19). In the Japanese cohort, 15 of 70 patients with IgAN had detectable serum anti-SPTBN1 IgA antibodies, whereas none were found in DC patients (sensitivity, 21%; specificity, 100%) (Figure 1a). In the UK cohort, 15 of 49 patients with IgAN had detectable anti-SPTBN1 IgA antibodies, whereas 3 of 19 patients with DC (1 patient with MN and 2 patients with antineutrophil cytoplasmic antibody–related vasculitis) had detectable levels (sensitivity, 31%; specificity, 84%) (Figure 1b). In total, 30 of 119 patients with IgAN and 3 of 51 patients had detectable serum anti-SPTBN1 IgA antibodies (sensitivity, 25%; specificity, 94%).

### Sensitivity and Specificity of Anti-CBX3 IgA Antibodies in Patients With IgAN

**Figure 2:**
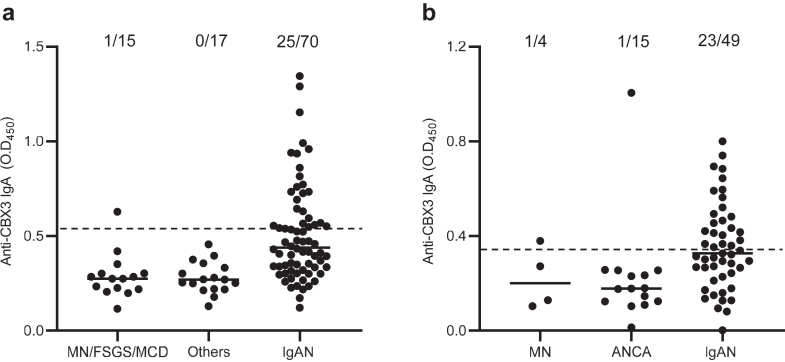
Detection of anti-CBX3 IgA antibodies in serum from patients with IgAN. Serum anti-CBX3 IgA antibody levels in patients with IgAN and DC (a) from the Japanese cohort and (b) from the UK cohort. Dashed lines show the cut-off value. Each dot represents the optical density (O.D.) value of a patient and the horizontal lines represent the medians. The numbers above the dots represent the overall number of patients with each disease and the number of patients with anti-CBX3 IgA antibodies. ANCA, antineutrophil cytoplasmic antibody–related vasculitis; DC, disease control; FSGS, focal segmental glomerulosclerosis; IgAN, IgA nephropathy; MN, membranous nephropathy; MCD, minimal change disease; Others, other kidney diseases.

**Figure 3:**
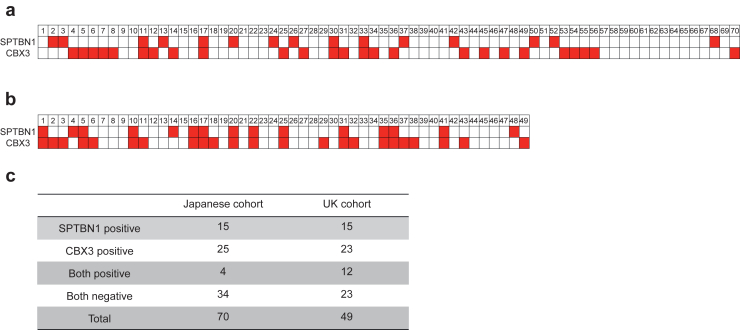
Summary of the presence of antimesangium IgA antibodies in our cohort. (a) Figure showing the presence of anti-SPTBN1 and anti-CBX3 IgA antibodies in each patient with IgAN (a) from the Japanese and (b) the UK cohort. The red-checked box indicates presence of anti-SPTBN1 or anti-CBX3 IgA antibody. The number at the top of the figure shows the patient's ID number. (c) The number of patients who were positive for anti-SPTBN1 IgA antibodies, anti-CBX3 IgA antibodies and both antibodies and negative for both antibodies are shown. IgAN, IgA nephropathy; SPTBN1, β2-spectrin.

In the Japanese cohort, 25 of 70 patients with IgAN had detectable serum anti-CBX3 IgA antibodies, whereas 1 (MN) of 32 DC patients had detectable levels (sensitivity, 36%; specificity, 97%) (Figure 2a). In the UK cohort, 23 of 49 patients with IgAN had detectable anti-CBX3 IgA antibodies, whereas 2 (MN = 1, antineutrophil cytoplasmic antibody–associated vasculitis = 1) of 19 DC patients had detectable levels (sensitivity, 47%; specificity, 90%) (Figure 2b). In total, 48 of 119 patients with IgAN and 3 of 51 DC patients had detectable serum anti-CBX3 IgA antibodies (sensitivity, 40%; specificity, 94%). Among the 70 Japanese patients with IgAN, 4 (6%) had detectable serum anti-SPTBN1 and anti-CBX3 IgA antibodies, and 32 (46%) had detectable levels of one or the other. Among the 49 UK patients with IgAN, 12 (25%) had detectable serum anti-SPTBN1 and anti-CBX3 IgA antibodies, and 14 (29%) had detectable levels of one or the other. In total, 62 patients with IgAN (52%) had detectable serum anti-SPTBN1 and/or anti-CBX3 IgA antibodies (Figure 3).

### Characteristics of Patients With IgAN With Antimesangium IgA Antibodies

Full clinicopathological data were available in 95 patients with IgAN (Japan = 70, UK = 25). The patients were divided into the following 4 groups: presence of serum anti-SPTBN1 IgA antibodies (*n* = 23), presence of serum anti-CBX3 IgA antibodies (*n* = 37), presence of both IgA antibodies (*n* = 10), and absence of both IgA antibodies (*n* = 45). Clinicopathological characteristics of the groups were compared (Table 1). Serum anti-SPTBN1 and anti-CBX3 IgA antibodies were more frequently detected in male patients with IgAN (*P* = 0.0047). There were no significant differences in estimated glomerular filtration rate or proteinuria between the groups. Serum Gd-IgA1 levels tended to be higher in patients with anti-SPTBN1 and anti-CBX3 IgA antibodies (*P* = 0.055). No significant pathological differences were observed between the presence or absence of antimesangium IgA antibodies. There was no difference in the degree of glomerular Ig (IgG and IgM) and complement C3 deposition in patients with IgAN and without antimesangium IgA antibodies (data not shown).

**Table 1:** Characteristics of patients with IgAN with antimesangium IgA antibodies

Characteristics	Anti-SPTBN1 IgA (*n* = 23)	Anti-CBX3 IgA (*n* = 37)	Both positive (*n* = 10)	Both negative (*n* = 45)	*P-*value
Clinical findings					
Female sex (%, [*n*])	30.4 [7]	27.0 [10]	20.0 [2]	60.0 [27]	0.0047
Laboratory findings					
eGFR (ml/min per 1.73 m^2^)	90.0 [70.2–104.1]	85.0 [66.6–95.8]	87.5 [83.5–95.8]	80.3 [61.0–95.7]	0.55
Proteinuria (g/d)	0.23 [0–0.40]	0.46 [0.20–1.00]	0.32 [0.02–0.49]	0.30 [0.06–0.86]	0.73
Serum Gd-IgA1 (μg/ml)	7.3 [4.9–8.3]	7.5 [5.0–8.9]	8.4 [7.8–9.2]	4.5 [3.5–6.3]	0.055
Pathological findings (Oxford MEST-C classification)					
M1 (%, [*n*])	47.8 [11]	59.5 [22]	70.0 [7]	33.3 [15]	0.36
E1 (%, [*n*])	34.8 [8]	27.0 [10]	20.0 [2]	31.1 [14]	0.24
S1 (%, [*n*])	65.2 [15]	64.9 [24]	60.0 [6]	57.8 [26]	0.15
T1+2 (%, [*n*])	8.7 [2]	24.3 [9]	10.0 [1]	22.2 [10]	0.27
C1+2 (%, [*n*])	8.7 [2]	24.3 [9]	0 [0]	28.9 [13]	0.18

eGFR, estimated glomerular filtration rate; Gd-IgA1, galactose-deficient IgA1; IgAN, IgA nephropathy; SPTBN1, β2-spectrin.

Clinicopathological characteristics of the following groups were compared: presence of serum anti-SPTBN1 IgA antibodies (Anti-SPTBN1 IgA, *n* = 23), presence of serum anti-CBX3 IgA antibodies (Anti-CBX3 IgA, *n* = 37), presence of both IgA antibodies (both positive, *n* = 10), and absence of both IgA antibodies (both negative, *n* = 45).. Categorical variables are presented as numbers (percentages); values for continuous variables are presented as median [interquartile range] or mean ± SD.

### SPTBN1 and CBX3 are Expressed on the Surface of Human Mesangial Cells

**Figure 4:**
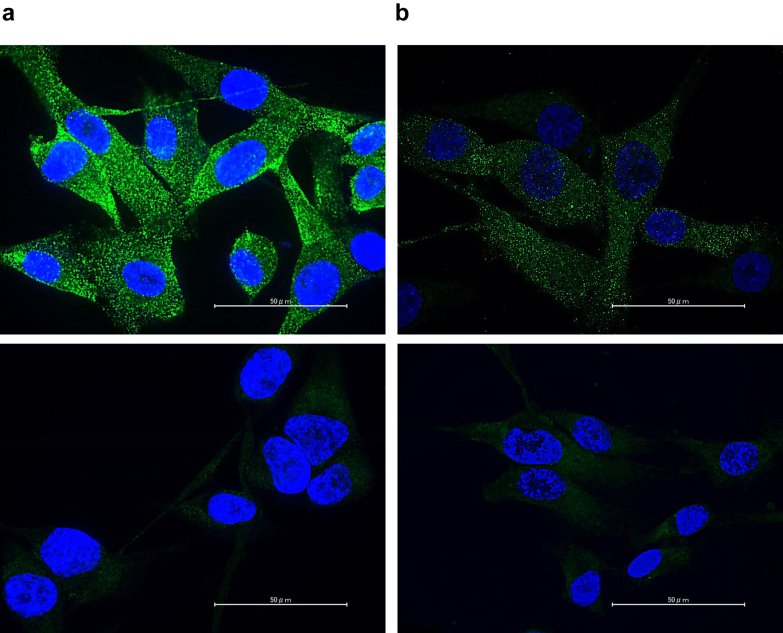
IF study showing surface expression of β2-spectrin and CBX3 on human mesangial cells. Representative IF microscopic images of surface staining of primary human mesangial cells are shown. Primary human mesangial cells were surface stained with rabbit (a) anti-β2-spectrin or (b) anti-CBX3 IgG antibody followed by Alexa Fluor 488-antirabbit IgG antibody (green) and 4abbit IgG antibody (green) a(blue) (top). Images of primary human mesangial cells stained with the secondary antibody alone (control) are also shown (bottom). The scale bars are shown as white lines (50 μm). IF, immunofluorescence.

We previously showed that SPTBN1 and CBX3 are expressed on the surface of murine mesangial cells.^13^^,^^14^ Thus, we investigated whether these proteins are expressed on the surface of human mesangial cells by IF staining. First, we examined whether our staining method (fixation with PFA followed by immunostaining with antibodies) detects intracellular molecules. In our previous studies, we found by flow cytometry that approximately 20% to 30% of HEK293T cells express SPTBN1 on their surfaces.^13^ We performed the staining method and approximately 25% of PFA-fixed HEK293T cells were immunostained with anti-SPTBN1 antibody (Supplementary Figure S1), which corresponds to discovery by flow cytometry. Conversely, almost no surface expression of CBX3 was observed on HEK293T cells by flow cytometry (Supplementary Figure S2). Consistent with this, PFA-fixed HEK293T cells were rarely immunostained with anti-CBX3 antibody (Supplementary Figure S3). These data demonstrated that our fixation method using PFA did not damage the integrity of the cell membranes, expose intracellular proteins and allow antibodies to immunostain only cell-surface proteins. Using this staining method, we immunostained primary human mesangial cells and demonstrated cell-surface expression of SPTBN1 and CBX3 (Figure 4).

### Anti-SPTBN1 and CBX3 IgA Antibodies are Enriched for Poorly Galactosylated IgA1 *O*-Glycoforms

**Figure 5:**
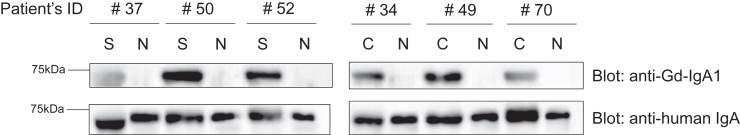
Anti-SPTBN1 and anti-CBX3 IgA antibodies were enriched for poorly galactosylated IgA1 *O*-glycoforms. Anti-SPTBN1 and CBX3 IgA antibodies were affinity-purified from sera of patients with IgAN with the indicated ID. Patient IDs are the same as those listed in Figure 3. 4 ng of the purified IgA (anti-SPTBN1 (S) or anti-CBX3 (C) IgA) and nonpurified sera (N) containing the same amount of IgA from the same patient were immunoblotted with anti-Gd-IgA1 antibody (KM55, top) or antihuman IgA antibody (bottom). Gd-IgA1, galactose-deficient; IgAN, IgA nephropathy; SPTBN1, β2-spectrin.

To determine the pattern of *O*-glycosylation of anti-SPTBN1 and anti-CBX3 IgA antibodies, serum anti-SPTBN1 and anti-CBX3 IgA antibodies were purified and poorly galactosylated IgA1 *O*-glycoforms were detected by Western blotting with an anti-Gd-IgA1 antibody (KM55). Anti-SPTBN1 and anti-CBX3 IgA antibodies were markedly enriched for poorly galactosylated IgA1 *O*-glycoforms compared with total serum IgA from the same patients (Figure 5).

## Discussion

In this study, we demonstrated the presence of anti-SPTBN1 and anti-CBX3 IgA antibodies in patients with IgAN from Japan and the UK. These IgA antibodies were detected in the serum of patients with IgAN with high specificity (94%). SPTBN1 and CBX3 were expressed on the surface of human mesangial cells according to IF microscopy. Importantly, anti-SPTBN1 and anti-CBX3 IgA antibodies were enriched for poorly galactosylated IgA1 *O*-glycoforms, which is consistent with the well-documented increase in these *O*-glycoforms in multiple ethnically diverse IgAN populations.^19^

Our data showed that whereas serum anti-SPTBN1 and anti-CBX3 IgA antibodies were uncommonly seen in other kidney diseases, they were not found in all patients with IgAN, either from Japan or the UK. One possible reason is that there may be other mesangial reactive IgA antibodies in IgAN where the antigen is yet to be elucidated. Our previous screening of human mesangial cell lysates with patient sera suggested that other antigens may exist.^13^ The identification of the first antigen in MN in 2009^3^ was followed by the discovery of over 10 new antigens over the proceeding decade^20^; thus, we expect that other mesangial antigens are likely to be identified in the future. Another explanation for the relatively low frequency of anti-SPTBN1 and anti-CBX3 IgA antibodies in our IgAN cohorts is that our detection method was insensitive. We previously showed that using denatured SPTBN1 in a Western blot is more sensitive than enzyme-linked immunosorbent assay for detecting anti-SPTBN1 IgA antibodies,^13^ suggesting that serum anti-SPTBN1 IgA antibodies are more likely to recognize SPTBN1 that differs from its original structure *in vivo*. Thus, determination of the epitopes recognized by anti-SPTBN1 and anti-CBX3 IgA antibodies, and the establishment of validated measurement methods for these IgA antibodies will be required for more broader clinical implementation.

We have confirmed that anti-SPTBN1 and anti-CBX3 IgA antibodies in the sera of patients with IgAN are enriched for poorly galactosylated IgA1 *O*-glycoforms; and therefore, contribute to the increased total Gd-IgA1 pool that has been widely reported in multiple IgAN cohorts.^19^ The fact that Gd-IgA1 is not elevated in the serum of all patients with IgAN^21^ and that serum Gd-IgA1 levels are also elevated in the blood of relatives of patients with IgAN who do not develop the disease^22^ suggests that the antigen specificity of the Gd-IgA1 may determine pathogenicity. In the cohorts studied, the titers of serum antimesangium IgA antibodies did not correlate with the estimated glomerular filtration rate and proteinuria measured at the same time (data not shown). This indicates that antimesangium IgA antibodies may be involved in the initiation of IgAN and that other factors determine the severity of the disease.

SPTBN1 and CBX3 are expressed by most nucleated cells in a variety of tissues^23^^,^^24^; therefore, staining kidney tissue with currently available antihuman-SPTBN1 or anti-CBX3 antibodies results in generalized tissue staining, both in health and disease. Thus, it is not feasible to determine whether these proteins are expressed on the surface of human mesangial cells using kidney biopsy specimens. Therefore, we stained cultured primary human mesangial cells and confirmed that both proteins are expressed on the mesangial cell surface. However, currently, it remains unclear why cell surface expression of these proteins is upregulated in mesangial cells.

Several aspects were not investigated in the present study. First, the study only included patients with IgAN from Japan and the UK. Therefore, validation using a larger group of patients from other countries is required. Second, further experiments are required to determine why these antimesangium IgA antibodies are exclusively IgA and not IgG or IgM.^13^ We recently showed that anti-CBX3 IgA antibodies are induced by oral bacteria in gddY mice through a mechanism of molecular mimicry^14^; namely, antimesangial IgA antibodies generated at mucosal surfaces to prevalent microbial pathogens may cross-react with mesangial antigens. Because these antibodies are generated within the mucosal associated lymphoid tissue, they will have a mucosal phenotype and will inherently be more likely to be enriched for Gd-IgA1, the exact phenotype of pathogenic IgA described by multiple investigators in IgAN.^1^ Finally, the pathogenicity of anti-SPTBN1 and anti-CBX3 IgA antibodies should be directly investigated in the future. Clinical studies to determine whether serum antimesangium IgA antibodies decrease with disease activity and increase with recurrence of IgAN in renal transplant recipients, and basic studies using animal models, are required to conclude that antimesangium IgA antibodies are involved in the pathogenesis of IgAN.

Collectively, we show that anti-SPTBN1 and anti-CBX3 IgA antibodies are detected with high specificity in patients with IgAN and enriched for IgA1 with poorly galactosylated *O*-glycoforms. Further validation is needed. Given the high specificity of anti-SPTBN1 and anti-CBX3 IgA antibodies for IgAN, the detection of antimesangium IgA antibodies could in the future allow early diagnosis of IgAN, and facilitate earlier intervention with ≥ 1 of the newly approved IgAN therapies, thereby reducing the lifetime risk of kidney failure for patients with IgAN.

## Disclosure

All the authors declared no competing interests.

## Supplementary Material

Supplementary File (PDF) **Supplementary Methods. Figure S1.** IF microscopic images of staining of HEK293T cells with anti-β2 spectrin antibody. **Figure S2.** Flow cytometric analysis of HEK293T cells stained with anti-CBX3 antibody. **Figure S3.** IF microscopic images of staining of HEK293T cells with anti-CBX3 antibody. **Table S1.** Characteristics of the patients with DC in our cohort.

## Footnotes

Supplementary File (PDF)

**Supplementary Methods.**
**Figure S1.** IF microscopic images of staining of HEK293T cells with anti-β2 spectrin antibody. **Figure S2.** Flow cytometric analysis of HEK293T cells stained with anti-CBX3 antibody. **Figure S3.** IF microscopic images of staining of HEK293T cells with anti-CBX3 antibody. **Table S1.** Characteristics of the patients with DC in our cohort.
