# Supplementary material for: The Prevalence and Characteristics of IgA Antibodies to β2-Spectrin and CBX3 in Immunoglobulin A Nephropathy
Source: Kidney Int Rep. 2025 Mar 3;10(5):1486–94. doi: 10.1016/j.ekir.2025.02.025 (PMC12142598; doi:10.1016/j.ekir.2025.02.025)
Supplement: Supplementary File (PDF) — Supplementary Methods. Figure S1. IF microscopic images of staining of HEK293T cells with anti-β2 spectrin antibody. Figure S2. Flow cytometric analysis of HEK293T cells stained with anti-CBX3 antibody. Figure S3. IF microscopic images of staining of HEK293T cells with anti-CBX3 antibody. Table S1. Characteristics of the patients with DC in our cohort. [file mmc1.pdf]

Supplementary Material

Supplementary Methods

Flow cytometry

HEK 293T cells were surface and intracellular stained with anti CBX3 IgG antibody followed by PE conjugated-anti-rabbit IgG antibody. Intracellular CBX3 were stained using a Foxp3 Staining Buffer Set (eBio-science) according to the manufacturer's protocol. Cells were stained with Fixable Viability Dye eFluorTM 506 (eBioscience) to exclude dead cells. All samples were analyzed using a FACS Celesta (BD Biosciences).

Supplementary Figure S1. IF microscopic images of staining of HEK293T cells with anti-β2 spectrin antibody

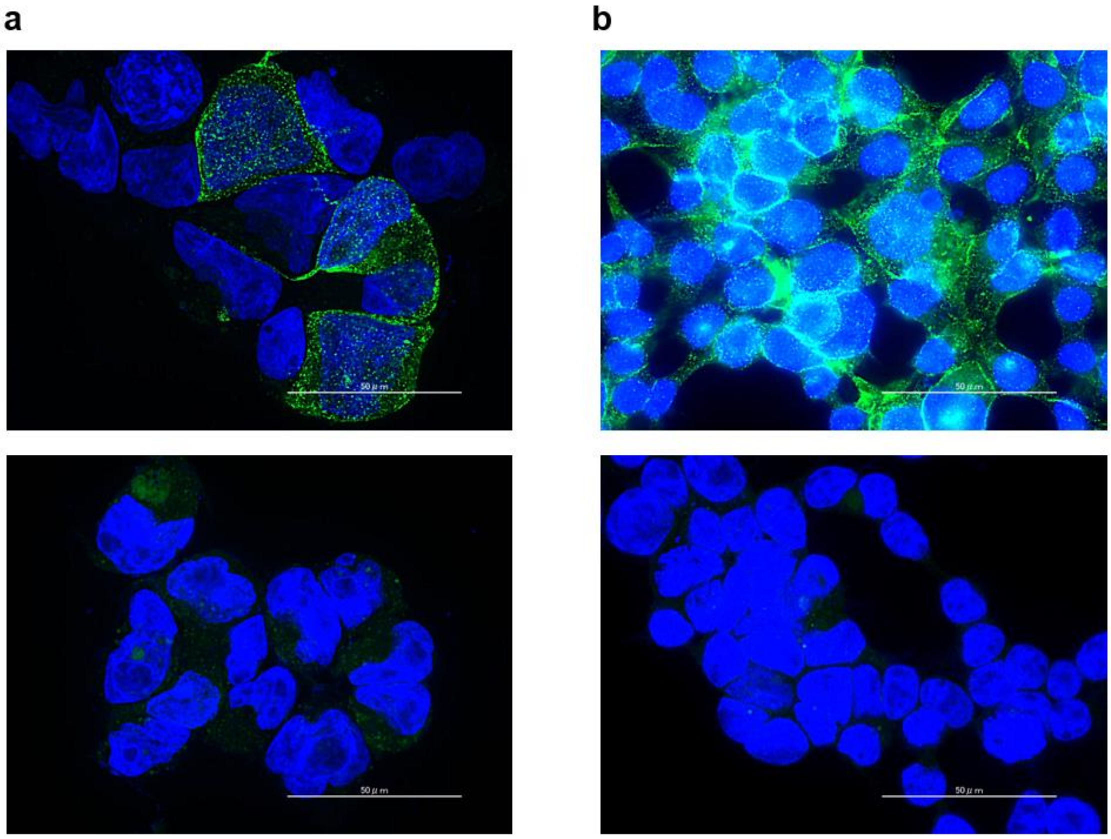

Representative IF microscopic images of staining of HEK293T cells are shown. PFA-fixed HEK293T cells were surface **(a)** and intracellular **(b)** stained with rabbit anti-β2 spectrin IgG antibody followed by Alexa Fluor 488-anti-rabbit IgG antibody (green) and DAPI (blue) (top). Images of HEK 293T cells stained with the secondary antibody alone (control) are also shown (bottom). The scale bars are shown as white lines (50 μm).

Supplementary Figure S2. Flow cytometric analysis of HEK293T cells stained with anti-CBX3 antibody.

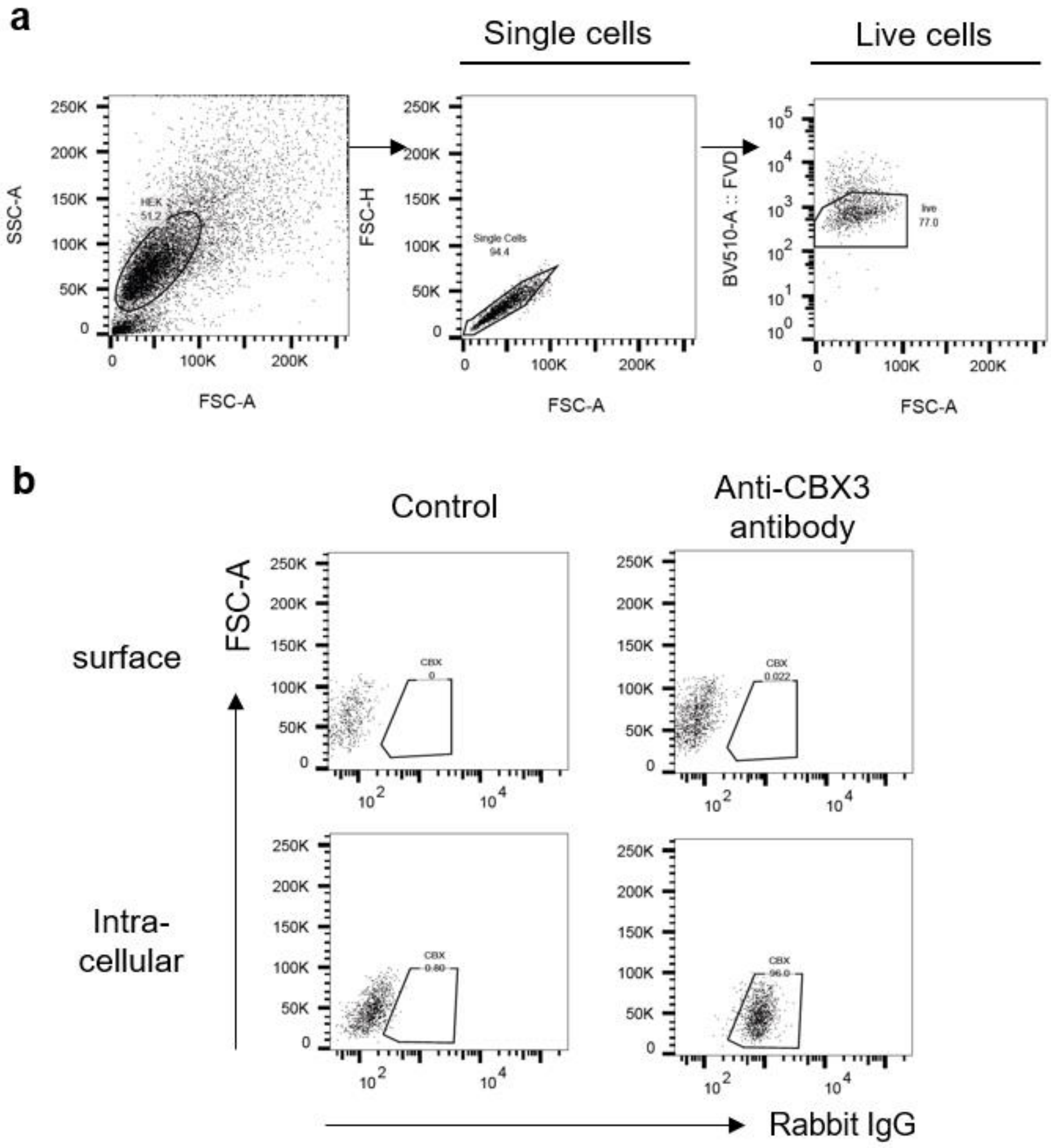

(a, b) Flow cytometric analysis of HEK293T cells. **(a)** Gating strategy of HEK293T cells. **(b)** Flow cytometry analyzing the surface (top) or intracellular (bottom) expression of

CBX3 on HEK293T cells gated on live cells. The cells stained with the secondary antibody alone (control) are also shown (top and bottom left).

Supplementary Figure S3. IF microscopic images of staining of HEK293T cells with anti-CBX3 antibody.

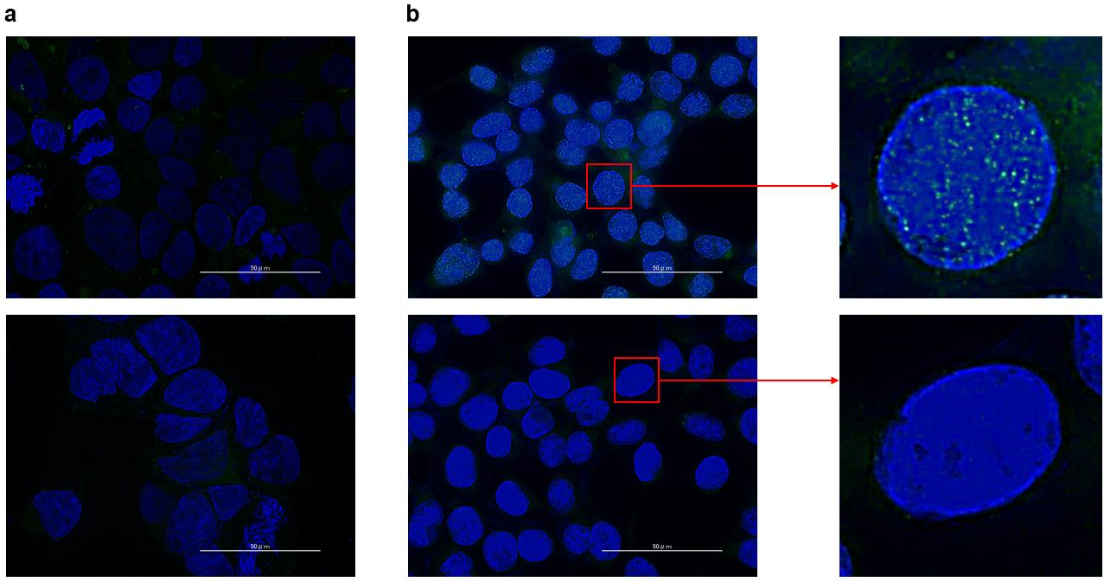

Representative IF microscopic images of staining of HEK293T cells are shown. HEK293T cells were surface **(a)** or intracellular **(b)** stained with rabbit anti-CBX3 IgG antibody followed by Alexa Fluor 488-anti-rabbit IgG antibody (green) and DAPI (blue) (top). Images of HEK 293T cells stained with the secondary antibody alone (control) are also shown (bottom). The scale bars are shown as white lines (50 μm).

Supplementary Table S1. Characteristics of the patients with DC in our cohort

ID	Cohort	Age	Sex	eGFR ($mL/min/1.73m^2$)	Proteinuria (g/day)	Diagnosis
DC1	Japan	21	Female	116.7	5.9	Minimal change disease
DC2	Japan	32	Male	101.2	2.9	Minimal change disease
DC3	Japan	32	Female	91.1	4.4	Minimal change disease
DC4	Japan	37	Female	94.1	unavailable	Minimal change disease
DC5	Japan	29	Male	59.4	4.1	Minimal change disease
DC6	Japan	35	Male	68.4	1.6	Focal segmental glomerulosclerosis
DC7	Japan	41	Female	47.8	1.14	Focal segmental glomerulosclerosis
DC8	Japan	27	Female	70.1	4.67	Focal segmental glomerulosclerosis
DC9	Japan	27	Female	52.2	0.96	Focal segmental glomerulosclerosis
DC10	Japan	33	Male	92.0	1.74	Focal segmental glomerulosclerosis
DC11	Japan	59	Male	58.9	3.5	Focal segmental glomerulosclerosis
DC12	Japan	49	Male	117.4	0.95	Membranous nephropathy
DC13	Japan	55	Male	90.7	4.24	Membranous nephropathy
DC14	Japan	54	Male	60.4	5.45	Membranous nephropathy
DC15	Japan	59	Female	75.0	0.45	Membranous nephropathy
DC16	Japan	50	Female	86.3	N.D.	Thin basement membrane disease
DC17	Japan	33	Male	79.8	N.D.	Thin basement membrane disease
DC18	Japan	27	Male	93.6	N.D	Thin basement membrane disease
DC19	Japan	32	Female	106.2	N.D.	Thin basement membrane disease
DC20	Japan	49	Male	48.1	N.D.	Thin basement membrane disease
DC21	Japan	61	Female	73.0	N.D.	Thin basement membrane disease
DC22	Japan	41	Male	69.9	N.D.	Thin basement membrane disease
DC23	Japan	49	Male	77.8	N.D.	Thin basement membrane disease
DC24	Japan	59	Male	64.0	N.D.	Thin basement membrane disease
DC25	Japan	52	Female	71.5	0.38	Alport syndrome
DC26	Japan	24	Male	91.9	3.05	Alport syndrome
DC27	Japan	23	Male	110.9	0.62	Alport syndrome
DC28	Japan	55	Male	63.5	N.D.	Alport syndrome
DC29	Japan	44	Male	25.2	0.69	Nephrosclerosis
DC30	Japan	60	Male	26.2	10.26	Diabetic kidney disease

DC31	Japan	56	Female	77.5	0.38	Non-IgA proliferative glomerulonephritis
DC32	Japan	53	Female	88.2	N.D.	Minor glomerular abnormalities
DC33	UK	unavailable	Male	unavailable	unavailable	Membranous nephropathy
DC34	UK	unavailable	Male	unavailable	unavailable	Membranous nephropathy
DC35	UK	unavailable	Male	unavailable	unavailable	Membranous nephropathy
DC36	UK	unavailable	Female	unavailable	unavailable	Membranous nephropathy
DC37	UK	unavailable	Male	unavailable	unavailable	ANCA associated vasculitis
DC38	UK	unavailable	Male	unavailable	unavailable	ANCA associated vasculitis
DC39	UK	unavailable	Male	unavailable	unavailable	ANCA associated vasculitis
DC40	UK	unavailable	Male	unavailable	unavailable	ANCA associated vasculitis
DC41	UK	unavailable	Male	unavailable	unavailable	ANCA associated vasculitis
DC42	UK	unavailable	Male	unavailable	unavailable	ANCA associated vasculitis
DC43	UK	unavailable	Male	unavailable	unavailable	ANCA associated vasculitis
DC44	UK	unavailable	Male	unavailable	unavailable	ANCA associated vasculitis
DC45	UK	unavailable	Male	unavailable	unavailable	ANCA associated vasculitis
DC46	UK	unavailable	Male	unavailable	unavailable	ANCA associated vasculitis
DC47	UK	unavailable	Male	unavailable	unavailable	ANCA associated vasculitis
DC48	UK	unavailable	Female	unavailable	unavailable	ANCA associated vasculitis
DC49	UK	unavailable	Female	unavailable	unavailable	ANCA associated vasculitis
DC50	UK	unavailable	Female	unavailable	unavailable	ANCA associated vasculitis
DC51	UK	unavailable	Female	unavailable	unavailable	ANCA associated vasculitis

ANCA, anti-neutrophil cytoplasmic antibody related vasculitis; eGFR, estimated glomerular filtration rate; N.D., not detected (less than 0.3g/gCr).
